# Validation of the instrument of health literacy competencies for Chinese-speaking health professionals

**DOI:** 10.1371/journal.pone.0172859

**Published:** 2017-03-06

**Authors:** Li-Chun Chang, Yu-Chi Chen, Li-Ling Liao, Fei Ling Wu, Pei-Lin Hsieh, Hsiao-Jung Chen

**Affiliations:** 1 School of Nursing, Chang Gung University of Science and Technology, Gui-Shan Town, Tao-Yuan County, Taiwan R.O.C; 2 Institute of Clinical Nursing, National Yang-Ming University, Taipei, Taiwan R.O.C; 3 Department of Health Management, I-Shou University, Kaohsiung City, Taiwan R.O.C; Universite de Bretagne Occidentale, FRANCE

## Abstract

The study aimed to illustrate the constructs and test the psychometric properties of an instrument of health literacy competencies (IOHLC) for health professionals. A multi-phase questionnaire development method was used to develop the scale. The categorization of the knowledge and practice domains achieved consensus through a modified Delphi process. To reduce the number of items, the 92-item IOHLC was psychometrically evaluated through internal consistency, Rasch modeling, and two-stage factor analysis. In total, 736 practitioners, including nurses, nurse practitioners, health educators, case managers, and dieticians completed the 92-item IOHLC online from May 2012 to January 2013. The final version of the IOHLC covered 9 knowledge items and 40 skill items containing 9 dimensions, with good model fit, and explaining 72% of total variance. All domains had acceptable internal consistency and discriminant validity. The tool in this study is the first to verify health literacy competencies rigorously. Moreover, through psychometric testing, the 49-item IOHLC demonstrates adequate reliability and validity. The IOHLC may serve as a reference for the theoretical and in-service training of Chinese-speaking individuals’ health literacy competencies.

## Introduction

The World Health Organization [1] defined health literacy as the cognitive and social skills that determine the motivation and ability of individuals to gain access to, understand, and use information in ways that promote and maintain good health. The important effects of an adequate level of health literacy on the disease management process have been observed in various studies [2–4] and drew the attention of health professionals towards successful care for people with chronic disease.

Several nationwide health literacy surveys have reported moderate to high prevalence of low health literacy (LHL) among adults, which is bound to result in healthcare problems [5–6]. Many reports have highlighted that low health literacy is associated with poor communication between patients and healthcare providers and with poor health outcomes, including increased hospitalization rates [7], poor medication adherence [4], and low effective self-care behavior [2]. The landmark report of the Institute of Medicine (IOM) [8] explicitly recommends that health professionals receive training in tailed patient education programs and in communicating effectively with patients with limited health literacy. Health education efforts could enhance people’s health literacy, thereby encouraging health-promoting behaviors [9].

Unfortunately, previous studies on health professionals have revealed insufficient knowledge of health literacy and misunderstanding of the contributions of health literacy towards patient health outcomes [10]. Health professionals’ limited ability to improve patients’ health literacy skills may affect patients’ capacity to better understand their illnesses and instructions on how to manage their health conditions [11].

Identification of key health literacy competencies among health professionals is fundamental for continued professional education. The education-based framework of a validated instrument could allow us to develop curricular interventions aimed at improving patients’ capacity for health literacy directly related to important self-care outcomes [12]. However, previous studies have yielded disappointing findings relating to health professionals’ health literacy knowledge or skills; these include the inability to correctly identify patients with LHL [13], lack of awareness of issues related to health literacy, including the impact of inadequate health literacy on patient care [14], no knowledge of health literacy resources, misconception of health literacy measurement, and discordance in the estimation of patients’ literacy levels [15].

There were differences in the self-reported use of various health literacy-oriented communication techniques among physicians, nurses, and pharmacists [16]. To date, the majority of measurements of health literacy competences have targeted physicians’ communication skills [17], with minimal emphasis on other core abilities related to the delivery of an educational program for other health professionals [18]. The first consensus study conducted by Coleman identified a comprehensive set of health literacy practices and measurable educational competencies (i.e., knowledge, skills, and attitudes) among students and health professionals. Further work is required to determine the suitability of all 94 items, and educational research is required to validate competencies in health literacy practice and to determine which should be taught, which healthcare professionals should receive training, which settings should be used, and which teaching methods should be adopted to improve patient-centered outcomes [12].

Conceptualizations of health literacy competence have been developed from the perspective of researchers, health professionals, and literacy experts, and using face-to face interviews with patients. These suggest that the categories of competencies in health literacy or other health communication constructs (e.g., cross-cultural communication, general interviewing skills, written communication, and shared decision making) are incomplete [12]. A scientific process for selecting suitable recommendations to include or exclude in the instrument to assess health professionals’ health literacy competencies is a crucial foundation for ensuring instrument validity. Thus, this paper describes the psychometric testing of the IOHLC, due to the importance of incorporating health literacy skills into healthcare providers’ education practices, as proclaimed by the WHO [19].

## Materials and methods

### Participants

This study formed part of a larger study aiming to develop an online competence module of health literacy for health professionals. In the current study, we report on the results of instrument development, which was conducted between May 2012 and January 2013. We invited three private Medical Centers in northern Taiwan and three private regional hospitals in southern Taiwan to participate in this study. Participants included non-physician healthcare professionals, case managers, health educators, dietitians, and pharmacists who had been working for at least six months. Participants who were expected to leave within a month were excluded from this study.

### Procedures

A convenience sample of 812 healthcare professionals completed self-administered online questionnaires. Information packets were distributed by hospital officials at selected sites to potential participants. The packets contained an information sheet, an informed consent form, and a reply envelope addressed to the primary investigator. Participants who agreed to participate in the study and returned the written inform consent forms received a link to the online questionnaires on the e-mail addresses that they provided. Completion of the online questionnaires took approximately 15–20 minutes. Online questionnaires were built into a learning platform and participants were required to use their e-mail addresses and date of birth to set the log-in profile. On this basis, we could ensure that participants personally responded to the questionnaire. Permission to conduct the study was obtained from the Institutional Review Board at Chang Gung Memorial Hospital (no: 100-4569B, approval date: 2012.3.16).

### Measure

A comprehensive set of measurable competencies of health literacy was established by using a modified Delphi technique with a panel of 24 experts who had experience in providing training on health literacy patient education [20]. Twenty-four experts (12 academic scholars in health literacy and 12 professionals with training related to health literacy practice) were invited to participate in the second to fourth rounds of data collection in the Delphi study. Moreover, consent forms were sent to the experts and returned to us prior to the commencement of our study.

The knowledge domain comprises 27 true/false items (12 were knowledge-specific items and 15 were related to attributes of patients with LHL). An incorrect answer for each item is assigned a score of 0 and a correct answer is assigned a score of 1. Three of the items (K1, K2, and A9) are reverse-worded and reverse-scored. The total score ranges from 0 to 27. Higher scores indicate higher levels of health literacy knowledge.

The skills domain consisted of 65 items related to health literacy practice. Respondents were required to indicate the level of agreement with items such as, “Being able to use plain language, instead of medical jargon.” A five-point Likert-scale was used for items in this domain, ranging from highly disagree (1) to highly agree (5). Higher scores indicated better health literacy skills.

The demographic data obtained from respondents included age, education level, occupational role at the clinic, years of experience in medicine/health care, and having ever heard of the term “health literacy.” We recruited 20 nurses for participation in the pilot survey before the investigation.

### Statistical analysis

#### Item response theory (IRT) analysis

Rasch analysis was used to assess the psychometric properties of the knowledge domain using the Andrich rating scale model [21] with Winsteps software (version 3.74.0) [22]. Two values are used throughout the analysis: logit measures and fit statistics. The logit (or log-odds units) is the natural logarithm of the odds of a participant succeeding at carrying out a specific task. Conventionally, 0 logit is ascribed to mean item difficulty. Moreover, the chi-square statistics for individual items’ (*p* > .05) difficulty and response ability (between +2.0 logits and -2.0 logits) were used as the determination criteria for the selection of items.

#### Exploratory factor analysis (calibration sample)

The validity of 65 items of the skills domain was established through exploratory factor analysis (EFA) and confirmatory factor analysis (CFA). The EFA and CFA were performed through use of a calibration sample and a validation sample, respectively. A total of 736 participants were equally divided into two subsamples, using the SPSS random case selection procedure (Version 15.0; SPSS Inc., Chicago, IL). The random selection procedures in SPSS were repeatedly performed until the homogeneity of baseline characteristics (e.g., education level, professional position, and tenure) among groups was warranted (*p* > .5). The first sample, termed the “calibration sample,” was used to explore the factor structure. In contrast, the second sample, termed the “validation sample,” was used to validate the factor structure constructed through the calibration sample.

With the calibration sample, principal component EFA was performed on the 65 items to extract the major contributing factors, and Varimax rotation was used to recognize relationships between items and common factors. The number of factors was decided upon, based on the eigenvalue-greater-than-1 rule. Factor loadings greater than 0.50 were regarded as significantly relevant and items with cross-loadings greater than 0.50 were deleted [23]. All item deletions were conducted one by one and the EFA model was re-specified following each deletion. The internal consistency of each construct was determined through Cronbach’s alpha; a value of 0.70 or higher was considered acceptable [24].

#### CFA (validation sample)

The validation sample was used to validate and modify the factor structure of the health literacy scale under development when the model did not fit the data. In the process of model modification, items that correlated too highly with others were deleted by examining the modification index (MI) of the additional specification of error covariance. A larger MI (e.g., >50) between two items indicated that those two items measured the same thing, thus necessitating deletion of one of the items, according to the parsimony principle [25]. The CFA model is to be modified until most of the model fit indices meet the criteria.

The goodness fit of the model was assessed using absolute fit indices, relative fit indices, and parsimony fit indices [23]. The absolute fit indices included the goodness of fit index (GFI), adjusted GFI index (AGFI), standardized root mean squared residual (SRMR), and the root mean square error of approximation (RMSEA). The relative fit indices were assessed through the normed fit index (NFI), non-normed fit index (NNFI), and the comparative fit index (CFI). Finally, the parsimony fit indices were determined using the parsimony normed fit index (PNFI), parsimony comparative fit index (PCFI), and the likelihood-ratio (χ2/df) [26].

The convergent validity of the items and factor structures was determined through standardized factor loading (values of 0.50 or higher were considered acceptable) and average variance extraction (AVE) (values of 0.50 or higher indicated acceptable) [23]. Convergent reliability was assessed through construct reliability (CR; values of .70 or higher were considered acceptable). The AVE and correlation matrices among the latent constructs were used to establish discriminant validity. The square root of the AVE of each construct was required to be higher than the correlation coefficient of that construct and other constructs [27].

## Results

### Participant characteristics

Among the 746 surveys returned from 6 hospitals, during analysis, we included 736 (99%) participants who had complete and valid data for all variables. All participants in this study were female. Table 1 describes the demographic characteristics of the study sample. The participants’ mean age was 30.71 ± 6.65 years (range = 20–60 years), with an average of 6.94 ± 6.25 years working in medicine/health care. The majority of participants (28.4%) had 1–3 years’ working experience, followed by those with 10–15 years’ (21.9%) experience. Participants’ professional roles at the hospital were registered nurse (78.5%), nurse practitioner (11.4%), dietician (4.5%), case manager (2.7%), health educator (2.4%), and health manager (0.5%) In total, 57.7% had a university education and 62.8% had never heard of the term, health literacy (see Table 1).

**Table 1:** Demographic Data for all Participants (*N* = 736).

Variables	*N*	%
Age (range: 20–60 years old)	*M* = 30.71	SD = 6.65
20–29	360	48.9
30–39	290	39.4
40–49	66	9.0
50–59	19	2.6
≥60	1	0.1
Years working in medicine/health care (6 m to 36 years)	*M* = 6.94	*SD* = 6.25
<1	37	5.0
1–3	208	28.4
4–6	157	21.4
7–9	102	13.7
10–15	161	21.9
>15	71	9.6
Role at clinic		
Registered nurse	578	78.5
Nurse practitioner	84	11.4
Health educator	17	2.4
Case manager	20	2.7
Dietician	33	4.5
Health manager	4	0.5
Education		
Graduate school	24	3.3
University	425	57.7
College	274	37.2
Nursing high school	13	1.8
Having heard of health literacy		
Yes	274	37.2
No	462	62.8

#### IRT calibration for 27 items of the knowledge domain

In the IRT analysis of the 27 items of the knowledge domain, 16 misfit items identified because of Rasch misfit statistics and measurement redundancy (*p* < .05) were deleted from the final questionnaire (see Table 2).

**Table 2:** Results of the Goodness-of-Fit Statistics for Knowledge Domain in the Rasch Model.

	Difficulty	Item goodness-of-fit		% of correct
Item (answer)		*χ*	*p*-value	
K1.Health literacy refers only to a person’s ability to read. (false)	1.60	146.27	< .001	12.9%
K2.Adequate health literacy is the ability to read, understand, and process health information. (false)	-2.85	10.48	.313	96.8%
K3.Those with low health literacy have poorer health outcomes relative to those with sufficient health literacy. (false)	**-2.22**	**21.09**	**.012**	93.4%
K4.Age is a risk factor that decreases health literacy. (true)	-0.35	16.10	.065	60.2%
K5.Patients with high educational levels may present with low health literacy. (true)	0.42	17.32	.054	37.6%
K6.Limited health literacy can produce barriers to clear, effective communication. (true)	-0.84	20.36	.016	73.1%
K7. Using an appropriate tool is the best way to assess health literacy and identify patients with low health literacy. (false)	-1.64	13.02	.162	37.6%
K8.People with low health literacy need extra medical support and therefore have higher healthcare costs. (true)	0.05	16.66	.054	48.5%
K9.Health education materials should be written at or below a seventh-grade reading level. (true)	-1.54	4.85	.847	86.3%
K10.Health literacy could affect physician-patient communication. (true)	-2.21	7.11	.626	93.3%
K11.Individuals with high educational levels also need an easy method of learning complicated health information. (true)	-2.23	6.89	.001	89.9%
K12.The general rule is to write consent documents at a seventh-grade reading level. (true)	-1.11	7.12	.001	90.2%
Patients with low health literacy …..				
A1. will say, “I can do this, there is no need to teach me” to cover up for their lack of understanding (true).	-0.68	9.29	.411	69.2%
A2. will repeat the same questions. (true)	-0.27	27.07	.001	58.0%
A3. will not tell you if they cannot read. (true)	-1.67	16.40	.059	88.0%
A4. are more likely to misinterpret medication instructions provided on prescription labels. (true)	-0.24	41.57	< .001	57.2%
A5. will easily misunderstand prescription instructions. (true)	-0.40	21.83	.009	61.6%
A6. cannot understand medication indications. (true)	-0.68	48.29	< .001	69.3%
A7. will often bring family members along when talking to healthcare professionals. (true)	-0.72	68.69	< .001	70.1%
A8. will make excuses to avoid reading health information materials when given material to read. (true)	-0.30	19.52	.021	58.7%
A9. often complain about their medicine. (false)	-0.29	50.27	< .001	58.5%
A10.only seek assistance when symptoms worsen. (true)	-0.25	49.17	< .001	57.5%
A11.cannot understand medical forms and are therefore unable to complete them accurately. (true)	-1.11	22.81	.007	79.1%
A12.are likely to put a lot of folded paper in their pockets or wallets. (true)	-0.95	68.23	< .001	75.7%
A13.do not make necessary appointments or attend follow up. (true)	-0.08	22.83	.007	52.5%
A14.may be likely to pose few questions to professionals. (true)	-0.56	82.80	< .001	66.1%
A15.cannot talk about how to take medicine. (true)	-0.45	25.18	.003	63.1%

*Note*. Discrimination = 1.19

The estimated item difficulty for retained items ranged from -2.00 logits (least difficult) to +2.64 logits (most difficult). Among the remaining nine items, seven were knowledge items (K2, K4, K5, K7, K8, K9, and K10) and two were LHL items (A1 and A3). The rate of correct answers for each item ranged from 37.6% to 96.8%.

#### EFA results for the calibration sample

EFA was performed sequentially a total of five times until none of the items factored less than .50 and there were no cross-loadings. In the first to the fifth step, four items were deleted, due to each factoring less than .50. No cross-loading was found in this phase, resulting in 61 remaining items that met the specified criteria.

The results obtained for descriptive statistics, EFA, and internal consistency (Cronbach’s α coefficient) are summarized in Table 3. The nine extracted factors represented 72.0% of the variance in the 61 items. These were “design teaching plan for LHL,” “simple and concrete teaching,” “build a friendly environment,” “use easy-to-use resources,” “life-oriented teaching,” “check for understanding,” “encourage clients to ask questions,” “self-designed materials to clients,” and “interdisciplinary collaboration.” Cronbach’s alphas greater than .80 were obtained for each subscale and that of the total scale was .97.

**Table 3:** Results of Descriptive Statistics, Exploratory Factor Analysis, and Reliability Analysis for Calibration Sample (*n* = 368).

			Factor loading								
No. Abbreviated Item Content	Mean	*SD*	1	2	3	4	5	6	7	8	9
Q11 Encourage clients to demonstrate learned skills to determine their understanding	3.84	0.78	.11	.20	.07	.08	.09	**.73**	.09	.12	.06
Q12 Ask clients to provide evidence of their health behavior	3.58	0.85	.21	.19	.00	.19	.05	**.65**	.23	.18	.14
Q13 Make eye contact with patients to ensure concentration	4.01	0.78	.08	.09	.19	.03	.15	**.78**	-.01	-.01	.06
Q14 Ask clients to restate the key points that they have learned	3.83	0.78	.14	.19	.12	.06	.09	**.72**	.11	.09	.02
Q15 Pay attention to questions that patients ask repeatedly	3.92	0.69	.11	.11	.16	.04	.08	**.76**	.11	.09	.10
Q16 Observe nonverbal (e.g., facial) expressions to determine whether the patient has understood	4.16	0.76	.06	.09	.25	.04	.25	**.72**	.00	-.10	.04
Q32 Handle the psychical barriers to conducting health behaviors for clients	3.56	0.73	.36	.17	.15	.15	.09	.17	.17	.22	**.63**
Q33 Cooperate with other professionals to design health education plans	3.58	0.76	.37	.19	.16	.13	.18	.11	.07	.17	**.65**
Q34 Design audio-visual teaching materials	3.23	0.92	.34	.16	.02	.28	.01	.04	.06	.44	**.55**
Q35 Have the language ability to handle different patients	3.61	0.81	.32	.10	.10	.10	.25	.15	.05	.13	**.68**
Q36 Cooperate with other professionals to implement behavior modification counseling	3.54	0.91	.39	.19	.02	.22	.16	.10	.05	.25	**.63**
Q38 Design computer-based teaching aids	3.18	0.90	.32	.19	.09	.18	.01	.09	-.01	**.67**	.24
Q39 Design health education flyers with less than 20% text	3.33	0.87	.39	.09	.19	.15	.13	.14	.04	**.65**	.13
Q40 Apply appropriate education theories in the curriculum	3.28	0.81	.49	.13	.21	.15	.23	.09	.11	**.52**	.18
Q41 Establish a personal profile of teaching materials	3.17	0.90	.48	.11	.13	.19	.11	.06	.04	**.62**	.20
Q42 Design a teaching plan for multicultural populations	2.75	0.99	.48	.00	.00	.23	.05	.04	.19	**.62**	.10
Q43 Design education materials for the illiterate	2.99	0.99	.43	.02	-.01	.23	.13	.09	.17	**.62**	.15
Q44 Determine the right teaching time for various clients	3.36	0.85	**.62**	.18	.20	.13	.20	.21	.07	.30	.18
Q45 Determine potential education barriers based on patient characteristics	3.40	0.77	**.67**	.19	.18	.12	.20	.13	.10	.23	.21
Q46 Apply appropriate tools to assess patient health literacy levels	3.37	0.82	**.73**	.14	.15	.15	.19	.12	.15	.21	.23
Q47 Conduct health assessments by collecting factors at personal, organizational, and community levels	3.31	0.84	**.73**	.20	.14	.16	.14	.08	.16	.21	.17
Q48 Identify the classical attributes of low health literacy prior to teaching	3.39	0.83	**.74**	.17	.14	.13	.16	.13	.11	.22	.12
Q49 Build up the right evaluation criteria for health literacy practice	3.35	0.79	**.77**	.24	.18	.19	.16	.08	.11	.17	.12
Q50 Conduct appropriate evaluations to demonstrate the effectiveness of health literacy practice	3.36	0.80	**.79**	.18	.16	.18	.16	.08	.17	.15	.17
Q51 Modify education plans to fit patients’ problems	3.45	0.80	**.79**	.21	.23	.15	.16	.11	.08	.11	.13
Q52 Illustrate the appropriate effectiveness of teaching based on health literacy	3.40	0.80	**.79**	.18	.18	.20	.15	.13	.15	.11	.16
Q53 Limit curricula to two or three new topics	3.38	0.80	**.79**	.15	.14	.22	.11	.13	.12	.15	.09
Q54 Design various evaluation approaches according to clients’ health literacy levels	3.33	0.89	**.74**	.18	.17	.25	.13	.10	.13	.14	.11
Q55 Use plain language instead of medical jargon	4.03	0.73	.17	.32	.26	-.02	**.58**	.13	-.05	.13	.06
Q57 Use metaphors to explain the disease to clients	3.82	0.73	.23	.23	.11	.09	**.66**	.23	.22	.14	.07
Q58 Use life-oriented examples to explain the care that patients need	3.95	0.71	.24	.27	.15	.09	**.76**	.18	.08	.12	.12
Q59 Teach using a language that the client understands	4.05	0.72	.20	.21	.32	.01	**.74**	.13	-.02	-.04	.12
Q60 Use materials available to educate the patient	3.96	0.73	.23	.25	.23	.02	**.71**	.14	.17	.03	.11
Q61 Connect new learning with previous experiences	3.77	0.73	.24	.25	.15	.04	**.62**	.13	.23	.15	.19
Q64 Teach repeatedly when clients cannot understand the teaching content	3.89	0.73	.23	**.54**	.29	.04	.32	.25	.13	.05	.04
Q66 Use the demonstrate-do technique	3.95	0.77	.16	**.64**	.12	.16	.19	.23	.14	.08	.03
Q67 Provide health education materials and encourage clients to discuss them with their families	3.90	0.78	.16	**.63**	.29	.07	.18	.17	.16	.11	.14
Q68 Provide health education materials with “Questions & Answers”	3.88	0.80	.26	**.68**	.27	.07	.08	.04	.13	.03	.14
Q69 Use simple words to explain care plans and related treatment	3.85	0.77	.18	**.71**	.26	.10	.29	.12	.16	.07	.14
Q70 Base decisions regarding teaching focus on treatment progress	3.76	0.77	.21	**.68**	.24	.23	.26	.14	.10	.12	.11
Q71 Summarize the key points of teaching at the end of the interview	3.68	0.82	.17	**.60**	.22	.32	.26	.19	.08	.17	.07
Q72 Instruct how to do rather than explaining the disease or condition	3.69	0.80	.26	**.60**	.26	.31	.15	.18	.08	.03	.15
Q73 Use pictorial methods, rather than words, to emphasize importance of issues for clients	3.40	0.98	.29	.40	.04	**.62**	.11	.11	.22	.08	.09
Q74 Provide self-designed sticks to allow clients to mark their records	3.01	1.05	.22	.17	.08	**.81**	-.01	.07	.20	.12	.08
Q75 Use the one-by-one method and pictorial image material	3.11	1.10	.24	.17	.15	**.80**	.02	.08	.17	.09	.13
Q76 Prepare teaching materials or teaching aids in health education	2.96	1.08	.24	.15	.18	**.82**	.06	.06	.15	.12	.10
Q77 Use media to benefit teaching outcomes	2.96	1.07	.21	.07	.17	**.78**	.04	.03	.23	.18	.11
Q78 Use online or Internet teaching	2.93	1.10	.20	.03	.23	**.75**	.02	.09	.18	.23	.09
Q79 Consider disobedient behavior to be temperate coping behavior	3.42	0.86	.20	.07	**.56**	.43	.15	.20	.13	.13	.10
Q80 Offer more encouragement to patients and illiterate clients	3.78	0.82	.18	.23	**.66**	.22	.24	.16	.03	.08	.18
Q81 Understand clients’ disobedient behaviors	3.75	0.81	.20	.25	**.64**	.22	.24	.16	.09	.10	.15
Q82 Invite caregivers to participate in the teaching plan	3.82	0.83	.20	.41	**.60**	.16	.14	.16	.14	.13	.18
Q83 Encourage clients and their families and clarify unclear parts of teaching via telephone	3.74	0.91	.25	.24	**.65**	.13	.09	.13	.29	.03	.02
Q84 Present oneself to clients as a resource	3.75	0.84	.21	.30	**.60**	.25	.12	.14	.25	.13	.04
Q85 Create an environment of mutual trust	3.88	0.80	.24	.32	**.63**	.16	.23	.19	.24	.05	.03
Q87 Create an embarrassment-free environment	3.86	0.83	.17	.29	**.67**	.12	.21	.18	.21	.11	.00
Q89 Encourage clients to take notes during interviews	3.51	1.01	.19	.22	.26	.29	.07	.08	**.67**	.06	.03
Q90 Teach clients to ask, “What is my main problem?”	3.42	1.02	.19	.21	.22	.34	.10	.13	**.77**	.11	.07
Q91 Teach clients to ask, “What do I need to do?”	3.48	0.97	.20	.14	.23	.30	.11	.15	**.81**	.08	.09
Q92 Teach clients to ask, “What can I do to help my body?”	3.49	0.95	.24	.13	.22	.30	.14	.12	**.80**	.06	.09
Q93 Encourage clients to talk about what doctors say to them	3.69	0.88	.17	.30	.38	.15	.25	.19	**.56**	.12	.10
Eigenvalue			9.6	5.9	5.5	5.5	4.4	4.3	3.9	3.5	2.9
Variance explained (%)			15.2	9.4	8.7	8.7	7.0	6.8	6.2	5.6	4.5
Cumulative variance (%)			15.2	24.6	33.2	41.9	48.9	55.7	61.9	67.5	72.0
Cronbach’s alpha			.97	.93	.93	.94	.91	.88	.94	.91	.89

*Note*. *SD* = Standard deviation

#### CFA results for the validation sample

As shown in Table 4, the result of the initial 61-item CFA revealed that none of the model fit indices met the criteria, with two exceptions, PNFI and PCFI. This indicated that the CFA model had to be modified. In the process of model modification, a total of 21 items were deleted sequentially (one by one) due to measuring constructs similar to those measured by other items. After the removal of 21 items, most of the model fit indices were acceptable, except for the GFI and the AGFI. Finally, the model fit indices of the modified model were generally acceptable, such that χ^2^/*df* = 1.90, NNFI = .94, CFI = .94, SRMR = .048, and RMSEA = .049 (see Table 4).

**Table 4:** Model Fit Indices of the Proposed Confirmatory Factor Analysis for Validation Sample (*n* = 368).

Global model fit	Acceptable criteria	Initial model (61 items)	Modified model (40 items)
Absolute fit index			
Likelihood-ratio χ^2^ (*p*-value)	*p* ≧ .05	4324.13 (< .001)	1334.94 (< .001)
GFI	≧ .90	.72	.85
AGFI	≧ .90	.69	.82
SRMR	≦ .05	.061	.048
RMSEA	≦ .05	.060	.049
Relative fit index			
NFI	≧ .90	.82	.90
NNFI	≧ .90	.88	.94
CFI	≧ .90	.89	.95
Parsimony fit index			
PNFI	≧ .50	.78	.81
PCFI	≧ .50	.84	.86
Likelihood-ratio χ^2^/*df*	≦ 2	2.33	1.90

*Note*. GFI = goodness fit index, AGFI = adjusted goodness fit index, SRMR = standardized root mean square residual, RMSEA = root mean square error of approximation, NFI = normed fit index, NNFI = non-normed fit index, CFI = comparative fit index, PNFI = parsimony normed fit index, PCFI = parsimony comparative fit index, *df* = degree of freedom.

As Table 5 illustrates, all standardised factor loadings exceeded the threshold of .50 and the AVE of each construct ranged from .58 to .82, which indicated excellent convergent validity. The construct reliability of all the constructs was greater than .70, which indicated acceptable convergent reliability.

**Table 5:** Factor Loading, Convergent Reliability and Convergent Validity of Confirmatory Factor Analysis for the Validation Sample (*n* = 368).

Construct/Item	Mean	*SD*	λ	*R*^2^	CR	AVE
**Factor 1 Design teaching plan for LHL**	**3.34**				.96	.72
Q44	3.29	0.88	.76	.58		
Q46	3.35	0.84	.85	.72		
Q48	3.32	0.84	.87	.75		
Q49	3.30	0.84	.90	.80		
Q51	3.43	0.83	.88	.78		
Q53	3.38	0.86	.86	.73		
Q54	3.34	0.88	.83	.70		
**Factor 2 Simple and practical teaching**	**3.83**				.92	.65
Q64	3.88	0.74	.81	.66		
Q66	3.92	0.81	.74	.54		
Q69	3.86	0.74	.79	.63		
Q71	3.76	0.78	.84	.71		
Q72	3.75	0.79	.84	.71		
**Factor 3 Building a friendly environment**	**3.84**				.89	.64
Q81	3.76	0.77	.81	.65		
Q82	3.82	0.78	.80	.63		
Q85	3.89	0.76	.84	.70		
Q87	3.87	0.86	.75	.56		
**Factor 4 Design of easy-to-use materials**	**3.05**				.91	.73
Q74	3.12	1.07	.87	.75		
Q75	3.15	1.07	.86	.74		
Q76	2.99	1.08	.89	.80		
Q78	2.95	1.13	.79	.62		
**Factor 5 Life-oriented teaching**	**3.99**				.89	.59
Q55	4.08	0.69	.70	.49		
Q58	4.00	0.73	.83	.69		
Q59	4.08	0.71	.78	.61		
Q61	3.81	0.77	.78	.61		
**Factor 6 Checking for understanding**	**3.95**				.87	.58
Q11	3.87	0.73	.69	.48		
Q13	3.99	0.80	.77	.60		
Q14	3.85	0.81	.76	.57		
Q15	3.93	0.75	.83	.69		
Q16	4.12	0.76	.76	.58		
**Factor 7 Encouraging questions**	**3.50**				.95	.82
Q90	3.41	0.98	.95	.89		
Q91	3.48	0.95	.97	.93		
Q92	3.48	0.96	.92	.85		
Q93	3.63	0.90	.78	.61		
**Factor 8 Self-designed low literacy materials to clients**	**3.19**				.92	.66
Q38	3.20	0.96	.76	.58		
Q39	3.32	0.92	.83	.69		
Q41	3.20	0.91	.86	.75		
Q43	3.02	0.98	.79	.63		
**Factor 9 Interdisciplinary collaboration**	**3.44**				**.87**	**.67**
Q33	3.57	0.81	.82	.67		
Q34	3.24	0.94	.79	.62		
Q36	3.51	0.85	.86	.73		

*Note*: λ = standardized factor loading, *R*^2^ = reliability of item (also called square multiple correlation, SMC), CR = construct (component/composite) reliability, AVE = average variance extraction, all factor loadings were statistically significant at *p* < .001.

As shown in Table 6, the majority of square roots of the AVE of each construct (values in the diagonal elements) were greater than the corresponding inter-construct correlations (values below the diagonal). This suggested that the result supported the discriminant validity of the IOHLC.

**Table 6:** Discriminant Validity Among Latent Variables of Confirmatory Factor Analysis for the Validation Sample (*n* = 368).

Construct	1	2	3	4	5	6	7	8	9
Factor 1	**.85**								
Factor 2	.57	**.80**							
Factor 3	.56	.82	**.80**						
Factor 4	.61	.59	.50	**.85**					
Factor 5	.56	.82	.74	.41	**.77**				
Factor 6	.51	.67	.62	.30	.65	**.76**			
Factor 7	.56	.61	.70	.68	.54	.40	**.91**		
Factor 8	.88	.46	.44	.65	.41	.45	.51	**.81**	
Factor 9	.84	.49	.47	.56	.50	.53	.49	.88	**.82**

*Note*. The value in diagonal element is the square root of AVE of each construct; all correlation coefficients were statistically significant at *p* < .001.

## Discussion

### Internal consistency reliability

The findings of this study showed that the IOHLC meets internal consistency reliability standards. After calibration for validation, the final version of the 49-item health literacy competencies also revealed acceptable internal reliability, with Cronbach’s alpha > .80.

### Psychometric properties of the instrument of health literacy competence

In this study, we used the Rasch model and two-stage factor analysis to verify the knowledge dimension and the skill dimension of the IOHLC. The IOHLC, which consisted of 9 knowledge items and 40 skill items making up 9 dimensions, was verified as having good convergent validity, explaining 72% of total variance. Therefore, this scale has a good theoretical basis and can be used to design courses to train professionals in health literacy.

The number of items in existing instruments measuring health literacy knowledge for health professionals ranges from 6 to 29; these items pertain to health literacy knowledge in general, and specifically to disadvantageous factors for vulnerable populations, prevalence of LHL, and consequences for various countries [27–28]. As knowledge and skills were measured in different domains, we adopted Rasch analysis, which entailed examining the adequacy of the 27 items in the knowledge domain, based on difficulty and item goodness-of-fit statistics (*p* > .05). However, no tools for health literacy competencies have been verified through IRT. There were limited opportunities for the comparison of the current results with others.

This study validated the IOHLC with 40 skill items related to patient education. According to Schulz and Nakamoto [29], the concepts, health literacy practices, and communication skills are difficult to distinguish. Literature positioning health literacy as communication skills considered health literacy practices as written and oral communication [30]. This study did not follow the categorization in the majority of previous studies on health literacy skills, and did not develop items based solely on the communication level.

Conversely, this study combined interview results relating to practices within patient education planning to inform categorization. This is an important contribution by this study in relation to the identification of health literacy competencies. The scale developed in this study is aimed at assessing the health literacy competencies of professionals, and professionals are expected to improve clients’ health literacy through patient education. Since health literacy is not only about communication skills, health literacy competencies should also include assessment and confirmation of comprehension [29]. Among the nine factors in this study, five were related to communication, while the other four included a supportive environment and assessment and confirmation of patient comprehension, which were also mentioned in literature [30]. In our scale, these concepts were operationalized into measurable items, to enable comprehensive representation of the health literacy competencies of health professionals.

Coleman et al. [12] initially identified a set of health literacy practices and educational competencies. The current study collected a wide range of items from literature and interviews results pertaining to the concept of health literacy. The scale consisted of 65 skill items before elimination, which were slightly more than the 59 items by Coleman (27 skill items + 32 practice items). The greatest difference between this study and that by Coleman et al. is that the latter divided health literacy competencies into potential educational competencies and potential practices. Although some items in this study included similar skill or practice items, the nine factors pertaining to skill items in this study were based on patient education planning. These skill items can be used as indices of learning competencies.

In terms of contributions to future application, not only can this scale be used comprehensively for regular assessment of health professionals’ health literacy competencies, but it can also be used to design health literacy training through the incorporation of contents into education goals. Unlike procedures in tools of health literacy practices, education, or competencies, this study adopted procedures that are similar to those in most scale construction research. However, the validation in this study may be used to inform inferences about the reliability and validity of tools relating to health literacy knowledge and skills mentioned in literature and used in practice.

### The descriptive results of the IOHLC

The items established in this study were similar to those in other studies. However, items that were scored differently yielded different results. In terms of acceptable scores on knowledge items, lower scores were obtained for “assessment of health literacy” and “extra support for patients with LHL.” Higher scores were obtained on items relating to the definition of health literacy and risk factors of LHL. This is similar to the results of a study conducted by Jukkala et al. [14] on the health literacy knowledge of professionals, wherein lower scores were obtained for LHL outcomes and assistance needed.

Among the skills, lower scores were obtained on items related to designing educational materials, such as educational materials for LHL, educational materials applicable to different groups, and Internet and multimedia educational materials. This is similar to the results of Cafiero’s [18] study on nurses, whereby audio-visual and computer-aided teaching tools were applied less frequently. This is also similar to the research results obtained in Howard, Jacobson, and Kripalani’s [31] study on physicians and in Mackert’s [32] study on pharmacists, wherein the application of drawing visual aids in LHL educational materials was relatively low. Schlichting et al. [33] found that providing LHL educational materials was a skill that was not frequently applied by physicians, nurse practitioners, dentists, and dental hygienists. Since the measurement methods and goals were not completely identical to those in previous studies, we could not determine whether the different results were due to differences in health literacy competencies. To ascertain this, future studies should further measure different health professionals’ health literacy competencies, using the IOHLC-PE.

### Limitations

This study continued research on the health literacy competencies of professionals, which were determined by consensus through the Delphi method. Then, IRT and two-stage factor analysis were employed to verify health literacy competence tools for professionals, which were oriented towards patient health education. There are no similar existing studies, as previous studies on health literacy competency were more dispersed and did not have a consistent framework for reference. Although this study developed the first structural tool for health literacy competency, it still has limitations relating to professional groups used, the sampling method, and language.

First, this study selected health professionals who are not medical doctors in Taiwan, used hospitals as the sampling unit, due to the limitations of time and resources, and questionnaires were recovered from nursing professionals, mostly. Thus, the health literacy competencies reflected in the results may be limited in their application to other professional groups. Secondly, due to convenience sampling, only hospitals in the southern and northern regions of Taiwan were included, and remote areas were not covered. Thus, the results cannot be generalized to different levels of hospitals and in different regions. Finally, the original scale developed in this study is in Chinese, and the items on health literacy competencies were developed based on practice content. Although it was validated through factor analysis after being translated into English and fitting the hypothesis, this scale may only be applicable to Chinese-speaking professionals.

## Conclusions

The tool in this study is the first to validate health literacy competencies in a rigorous way. Through factor analysis and IRT, 92 items were simplified into the 49-item IOHLC that is orientated toward patient education and has construct validity, including the knowledge level and 9 sub-dimensional skill domains. It provides future Chinese-speaking professionals with a framework and guidance for designing health literacy courses.

### Practice implications

Chinese society only recently embarked on health literacy research. Because LHL has an increasingly important influence on health, the education and training of professionals has to keep abreast with developments. The reliable and valid IOHLC may serve as a reference for theoretical and in-service training on health literacy competencies, with courses designed based on the scale’s framework. Directors of medical institutions can also use this tool to regularly assess the health literacy competencies of professionals, so as to ensure the quality of health care provided. Health literacy is closely related to patient education. Because actual practices of different healthcare professionals may differ, we recommend that future studies investigate different groups, in order to analyze the performance of this scale with use on different patient education professionals.

## References

[1] World Health Organization: Division of Health Promotion, Education and Communications Health Education and Health Promotion Unit Health promotion glossary. Geneva: World Health Organization; 1998.

[2] Heijmans M, Waverijn G, Rademakers J, van der Vaart R, Rijken M. Health literacy: functional, communicative and critical health literacy of chronic disease patients and their importance for self-management. Patient Educ Couns. 2015;98: 41–48. 10.1016/j.pec.2014.10.006 25455794

[3] Mantwill S, Schulz PJ. Low health literacy associated with higher medication costs in patients with type 2 diabetes mellitus: evidence from matched survey and health insurance data. Patient Educ Couns. 2015;98(15): 1625–1630. 10.1016/j.pec.2015.07.006 26198546

[4] Zhang NJ, Terry A, McHorney CA. Impact of health literacy on medication adherence: a systematic review and meta-analysis. Ann Pharmacother. 2014;48(6): 741–751. 10.1177/1060028014526562 24619949

[5] Carmona RH. Health literacy: a national priority. J Gen Intern Med. 2006;21(8): 803.

[6] Kutner M, Greenberg E, Jin Y, Paulsen C. The health literacy of America’s adults: results from the 2003 National Assessment of Adult Literacy (NCES 2006–483). Washington, DC: National Center for Education Statistics, US Department of Education; 2006.

[7] Berkman ND, Sheridan SL, Donahue KE, Halpern DJ, Crotty K. Low health literacy and health outcomes: an updated systematic review. Ann Intern Med. 2011;155(2): 97–107. 10.7326/0003-4819-155-2-201107190-00005 21768583

[8] Institute of Medicine. Health literacy: a prescription to end confusion. Washington, DC: National Academic Press; 2004.

[9] Nutbeam D. Health literacy as a public health goal: a challenge for contemporary health education and communication strategies into the 21st century. Health Promot Int. 2010;15(3), 259–267.

[10] Macabasco-O’Connell A, Fry-Bowers EK. Knowledge and perceptions of health literacy among nursing professionals. J Health Commun. 2011;16(3): 295–307. 21951259 10.1080/10810730.2011.604389

[11] Lambert M, Luke J, Downey B, Crengle S, Kelaher M, Reid S, et al Health literacy: health professionals’ understandings and their perceptions of barriers that Indigenous patients encounter. BMC Health Serv Res. 2014;14(1): 614. 25471387 10.1186/s12913-014-0614-1 PMC4267746

[12] Coleman CA, Hudson S, Maine LL. Health literacy practices and educational competencies for health professionals: a consensus study. J Health Commun. 2013;18(Suppl 1): 82–102. 24093348 10.1080/10810730.2013.829538 PMC3814998

[13] Ali NK, Ferguson RP, Mitha S. Do medical trainees feel confident communicating with low health literacy patients? J Community Hosp Intern Med. 2014;4(2). 10.3402/jchimp.v4.22893. 10.3402/jchimp.v4.22893 PMC3992362 24765262

[14] Jukkala A, Deupree JP, Graham S. Knowledge of limited health literacy at an academic health center. J Contin Educ Nurs. 2009;40(7): 298–302; quiz 303–294, 336. 19639850 10.3928/00220124-20090623-01

[15] Turner T, Cull WL, Bayldon B, Klass P, Sanders LM, Frintner MP, et al Pediatricians and health literacy: descriptive results from a national survey. Pediatrics. 2009;124(Suppl 3): S299–S305. 19861484 10.1542/peds.2009-1162F

[16] Schwartzberg JG, Cowett A, VanGeest J, Wolf MS. Communication techniques for patients with low health literacy: a survey of physicians, nurses, and pharmacists. Am J Health Behav. 2007;31(Suppl 1): S96–S104. 17931143 10.5555/ajhb.2007.31.supp.S96

[17] Coleman C. Teaching health care professionals about health literacy: a review of the literature. Nurs Outlook. 2011;59(2): 70–78. 10.1016/j.outlook.2010.12.004 21402202

[18] Cafiero M. Nurse practitioners’ knowledge, experience, and intention to use health literacy strategies in clinical practice. J Health Commun. 2013;18(Suppl 1): 70–81. 24093347 10.1080/10810730.2013.825665 PMC3815083

[19] Parnell TA. Health literacy in nursing: providing person-centered care. New York: Springer Publishing Co Inc; 2015.

[20] Chang LC, Chen YC, Wu FL, Liao LL. Exploring health literacy competencies toward health education program for healthcare professionals: a Delphi study. BMJ Open. 2016;10: e3120. 10.1136/bmjopen-2016-011772 PMC5253604 28093428

[21] Andrich D. A rating formulation for ordered response categories. Psychometrika. 1978;43(4): 561–573.

[22] Linacre JM. A user’s guide to WINSTEPS/MINISTEP: Rasch-model computer programs. 2009. Available from: http://www.winsteps.com/a/Winsteps-ManualPDF.zip).

[23] Hair JF, Black WC, Babin BJ, Anderson RE, Tatham RL. Multivariate data analysis. 6th ed. New Jersey: Prentice-Hall; 2006.

[24] DeVellis RF. Scale development: theory and applications. Newbury Park, CA: Sage; 1998.

[25] Chang WH. How to write and submit an academic paper with SEM. Taichung, Taiwan: Tingmao; 2011.

[26] Schumacker RE, Lomax RG. A beginner’s guide to structural equation modeling. 2nd ed. Mahwah, New Jersey: Lawrence Erlbaum Associates; 2004.

[27] Fornell C, Larcker D. Evaluating structural equation models with unobservable variables and measurement error. J Mark Res. 1981;18(1): 39–50.

[28] Pagels P, Kindratt T, Arnold D, Brandt J, Woodfin G, Gimpel N. Training family medicine residents in effective communication skills while utilizing promotoras as standardized patients in OSCEs: a health literacy curriculum. Int J Family Med. 2015;129187 10.1155/2015/129187 26491565 PMC4603603

[29] Schulz PJ, Nakamoto K. Health literacy and patient empowerment in health communication: the importance of separating conjoined twins. Patient Educ Couns. 2013;90(1): 4–11. 10.1016/j.pec.2012.09.006 23063359

[30] Peng J, Valpreda M, Lechelt K. Health literacy: improving communication between health care practitioners and older adults. Canadian Geriatrics Society Journal of CME. 2015;5(2): 1–30.

[31] Howard T, Jacobson KL, Kripalani S. Doctor talk: physicians’ use of clear verbal communication. J Health Commun. 2013;18(8): 991–1001. 10.1080/10810730.2012.757398 23577746

[32] Mackert M. Health literacy knowledge among direct-to-consumer pharmaceutical advertising professionals. Health Commun. 2011;26(6): 523–533. 10.1080/10410236.2011.556084 21469006

[33] Schlichting JA, Quinn MT, Heuer LJ, Schaefer CT, Drum ML, Chin MH. Provider perceptions of limited health literacy in community health centers. Patient Educ Couns. 2007;69(1–3): 114–120. 10.1016/j.pec.2007.08.003 17889494 PMC2246059

